# Psychometric properties of insomnia severity index in Iranian adolescents

**DOI:** 10.5935/1984-0063.20200045

**Published:** 2021

**Authors:** Azita Chehri, Nastaran Goldaste, Saman Ahmadi, Habibolah Khazaie, Amir Jalali

**Affiliations:** 1 Department of Psychology, Kermanshah Branch, Islamic Azad University, Kermanshah, Iran.; 2 Sleep Disorders Research Center, Kermanshah University of Medical Sciences, Kermanshah, Iran.; 3 Substance Abuse Prevention Research Center, Research Institute for Health, Kermanshah University of Medical Sciences, Kermanshah, Iran.

**Keywords:** Insomnia Severity Index, Reliability, Validation, Persian, Adolescent

## Abstract

**Objectives:**

Sleep deprivation and insomnia have negative impacts on mental and physical health in adolescents. In order to examine the problems caused by insomnia, we need tools with acceptable validity and reliability for the target population. The aim of this study was to evaluate the psychometric properties of Insomnia Severity Index (ISI) in Iranian adolescent population.

**Material and Methods:**

The study was carried out as a descriptive-analytic normalization. Study group consisted of 701 adolescents aged 12 to 19, who were selected through cluster sampling. The participants completed ISI, Epworth Sleepiness Scale (ESS), Pittsburgh Sleep Quality Inventory (PSQI), Adolescent sleep hygiene scale (ASHS) and global sleep assessment questionnaire (GSAQ). In addition, SPSS ver.22 and AMOSS ver.10 were used to analyze the data.

**Results:**

The reliability of the test was obtained equal to 0.77 based on Cronbach’s alpha and a good reliability (0.84) was obtained through test/retest method. Exploratory and conﬁrmatory factor analyses with seven items were computed. The models ﬁtness indexes were suitable for the structural model (CFI=0.99%, NFI=0.98%, GFI=0.99%, TLI=0.98%, RMSEA=0.049%). Moreover, there was a direct and signiﬁcant correlation between the ISI index and the total score of other indexes. As to gender-based reliability, Cronbach’s alpha was 0.78 in female adolescents and 0.85 in male adolescents.

**Conclusion:**

The results indicated that the Persian version of the insomnia severity index can be used as a reliable and valid tool for assessing the severity of insomnia in Persian-speaking adolescents.

## INTRODUCTION

Insomnia seriously affects mental and physical health^1^ and even in some cases sleep problems predict future physical and psychiatric disorders^2^. The term “insomnia” is widely used in medical texts. In general, it can be defined as a person complaining of having trouble sleeping^3^. Insomnia is a condition characterized by difficulty in initiating or maintaining sleep and it is associated with symptoms of irritability or fatigue after waking up. The prevalence of this disorder ranges from 10% to 20% and in chronic cases it is up to 50%^4^.

The minimum sleep requirement is different for different person. If this minimum level is not met, the symptoms of insomnia can lead to a disturbance in life and performance^1^. These symptoms include irritability, loss of focus, fatigue, daytime sleepiness, and temporary forgetfulness^5^. The symptoms associated with insomnia can interfere with creativity, judgment, and discernment abilities^1^. Insomnia is one of the issues that disturbs life and prevents individuals from performing activities in an optimum way^6^. Studies have reported the association of insomnia with depression, weakened immune system, and heart disease^7^^,^^8^. Due to the numerous complications that chronic insomnia has on different physical, psychological, social and spiritual dimensions of individuals^1^, it is necessary to study this condition in different individuals in order to determine the extent of the disorder, ways to treat it, and the necessary interventions^6^^,^^9^.

Studies have shown that sleep patterns change with age. Many changes in sleep, such as sleep duration, shorter nighttime sleep, more frequent napping during the day, more frequent night awakenings and finding it hard to go asleep afterwards, and shorter duration of slow-wave sleep happen from young age to middle age^10^^-^^12^. In this regard, the prevalence and causes of insomnia vary depending on changes in sleep patterns and the components at different ages. So that the prevalence and causes of insomnia are different in adolescents, adults, and the elderly^1^^,^^4^^,^^13^.

Adolescence is a unique period of life that is characterized by physical, psychological, and social changes^8^. Inadequate sleep has a great impact on teenagers’ behaviors and in most cases leads to bigger problems^1^. Insomnia is very common in adolescents and this disorder can be associated with depression and other psychiatric disorders. It is an independent risk factor for suicide and substance use in adolescents. Therefore, examining the disorder and treating its symptoms in early adolescence may reduce the risk of these side effects^9^. Studies have shown that excessive technology use is a common factor with a significant effect on sleep duration and prolonged sleep onset latency in adolescents, which may lead to several sleep problems^14^. Therefore, it is essential to understand and treat insomnia in adolescents^1^.

The ISI is one of the most important tools to examine and measure sleep quality^15^. It is a brief instrument to assess the severity of the both nighttime and daytime components of insomnia. It is available in several languages and used as a metric of treatment response in clinical researches^16^. The ISI has been studied and validated in different populations^17^^-^^20^. This scale was validated by Sadeghniiat et al.^18^ for adults in Iran. Given the importance of the issue, the present study is an attempt to examine the psychometric properties of this scale in the Iranian adolescents.

## MATERIAL AND METHODS

The study was carried out as an applied descriptive-analytic normalization study. The statistical population included all adolescents in Kermanshah city, Iran. The adolescents’ population in Kermanshah province was about 421,266 in 2017 (Director of the Registration Office of Kermanshah Province). The statistical sample included adolescents aged 12 to 19 years from five districts of the city and they were selected based on cluster method (n=720; in the analysis phase, only 701 cases were found). For this purpose, Kermanshah city was divided into 10 districts and five of them were randomly selected. Then, each one of the five districts were divided into six sections out which three sections were randomly selected and eligible adolescences were selected from these sections through convenient sampling method. The questionnaires were distributed among the adolescents at schools and sports clubs with the help of five psychologists. Before distributing the questionnaires, the research colleagues (second and third authors) provided a complete explanation of the purpose of the study and the questions of the questionnaires to the respondent adolescents. These colleagues were responsible for collecting the data. After explaining the goals of this research and assuring the participants of the confidentiality of their personal information, the questionnaires were distributed and completed by the adolescents. The research assistants helped the subjects in the case of any ambiguity in the questions. The time for completion of the questionnaires was about 30 minutes. The inclusion criteria were being 12 to 19 years old, willingness to participate in this study, and accurately completing the questionnaires. The exclusion criteria were having specified psychiatric disease affecting sleep or taking drug and medications that affected sleep quality. The adolescents completed ISI, ESS, PSQI, ASHS, and GSAQ questionnaires.

After completing the questionnaires, the reliability was measured using Cronbach’s alpha. The ISI was completed once more 4-6 weeks later to measure retest reliability of 10% of participants. Confirmatory and Exploratory Factor Analysis (EFA & CFA) were computed to examine concurrent validation, construct validation, and factor structure. The reliability and validity of the gender-based insomnia severity index were also examined.

### Insomnia severity index (ISI)

The insomnia severity index has seven questions. The total score is equal to the sum of the seven questions. The questionnaire assesses the severity of problems of falling asleep, staying asleep, waking up early, and satisfaction with sleep. This questionnaire evaluates the interference of insomnia in daytime functions and intensity of the worries caused by sleep problems. The questions are four alternative questions and total score ranges from zero to 28. Higher scores indicate higher insomnia severity^21^. The results showed that the Persian version of the ISI had an acceptable internal consistency (Cronbach’s alpha of 0.78). It was also shown that the Persian version of ISI had enough differentiation power to distinguish patients from healthy ones^18^.

### Epworth Sleepiness Scale (ESS)

The Epworth sleepiness scale (ESS) is a self-administered questionnaire that is routinely used to assess daytime sleepiness. This simple self-reporting scale is designed to evaluate daytime sleepiness. It consists of eight questions that examine the respondent in eight different relatively common situations in everyday life. All questions in this scale are scored from 0 to 3. A zero score indicates that the chance of sleepiness is high. A total score of 10 and over indicates extreme daytime sleepiness^22^. Researchers have concluded that the questionnaire is a valid and reliable tool for assessing daytime sleepiness and it can be used in the clinical population and other populations^23^.

### Pittsburgh Sleep Quality Index (PSQI)

The questionnaire is designed to measure sleep quality and help diagnosing those with poor sleep quality^24^. Validity and reliability of this questionnaire have been measured for Iranian populations (α=0.83 and correlation coefficient=0.88)^25^.

### Adolescent Sleep Hygiene Scale (ASHS)

The original adolescent sleep hygiene scale is a 32-item self-reporting scale developed by LeBourgeois. The Cronbach’s alpha was satisfactory for the total sleep hygiene index, and the internal consistency in several areas was lower than the recommended levels (Cronbach’s alpha between 0.37 and 0.74)^26^. Chehri et al.^27^ validated this questionnaire and showed that the Cronbach’s alpha was between 0.71 and 0.75, and the correlation for reliability was between 0.82 and 0.87.

### Global Sleep Assessment Questionnaire (GSAQ)

The global sleep assessment questionnaire consists of 11 questions that assess sleep behaviors based on a three-point scale from zero (behaviors that never happen) to two (behaviors that always happen). The higher the total score of the tool represents the higher the risk of experiencing sleep disturbance. The test-retest reliability of this questionnaire was in the range of 0.51 to 0.92 and its concurrent validity was favorable based on the clinical experts’ evaluation^28^.

### Data analysis

Two-dimensional tables, mean, and variance were used to describe demographic variables. Cronbach’s alpha coefficient and intraclass correlation coefficient (ICC) were used to calculate the reliability and internal reliability of the instrument. Spearman’s correlation coefficient was used to measure the correlation between scale items and concurrent validity given that the data distribution was not normal. Finally, exploratory and confirmatory factor analyses were used to confirm the construct validity. In order to use EFA to verify the construct validity, it was necessary to conduct two preliminary tests. Kaiser-Meyer-Olkin (KMO) sampling adequacy test with a value of 0.84 (chi-square=1795.78) was used to test the statistical assumptions for factor analysis. The significance level of the Bartlett test was 0.02% (*p*=0.001). This means that the null hypothesis was rejected and there was a significant relationship between the variables.

## RESULTS

Totally, 701 adolescents with mean age of 16.52 years participated in this study. The participants were 12 to 19 years old (SD: 2.06) and the average time of using electronic tools was 5.20 hours (SD: 2.05). In addition, 51.8% were female and 48.2% were male; 34.2% had high school education, 16.8% had a history of smoking (cigarette, hookah, etc.); and 11.7% of them had a history of illnesses. Totally, 81% of research units lived with their parents (Table 1). The mean and standard deviation of variables and insomnia severity were examined (Table 2).

**Table 1:** Distribution of relative and absolute frequency and mean and standard deviation of demographic variables in research units.

Variables		Frequency	Percent (%)
Gender	Female	363	51.8
	Male	338	48.2
History of smoking	Yes	118	16.8
	No	583	83.2
History of illness	Yes	82	11.7
	No	619	88.3
Level of Education	Sixth grade of elementary school	63	9
	First grade of middle school	157	22.4
	Second grade of middle school	240	34.2
	High school diploma	202	28.8
	College student	39	5.6
The status of life	Living with parents	568	81
	Living with mother	123	17.55
	Living with father	4	0.6
	Living with grand mother	6	0.85
	**Mean±SD**	**Min.**	**Max.**
Age (year)	16.52±2.06	12	19
Time to play with electronic tools (hours)	5.20(2.05)	0.17	12

**Table 2:** Mean and standard deviation and Correlation coefficients matrix of ISI in research units.

Variable	Mean ± SD	Q1 R P-value	Q2 R P-value	Q3 R P-value	Q4 R P-value	Q5 R P-value	Q6 R P-value	Q7 R P-value	Total Score R P-value
Difficulty in falling asleep	0.98±0.96	1							
Difficulty in staying asleep	1±1.08	0.573** 0.001	1						
Difficulty in waking up early	0.98±1.63	0.356** 0.001	0.541** 0.001	1					
Satisfied / dissatisfied with recent pattern of sleep	0.98±1.63	0.521** 0.001	0.499 ** 0.001	0.440** 0.001	1				
Daytime dysfunction	1.08±1.43	0.488 ** 0.001	0.421** 0.001	0.383** 0.001	0.554** 0.001	1			
Significant loss of quality of life	1.14±1.06	0.308 ** 0.001	0.276** 0.001	0.279** 0.001	0.248** 0.001	0.445** 0.001	1		
Concern about sleep problem	1.14±1.06	0.473** 0.001	0.435** 0.001	0.408 ** 0.001	0.563** 0.001	0.558** 0.001	0.504** 0.001	1	
Severity of Insomnia	5.35±8.85	0.727** 0.001	0.720** 0.001	0.680** 0.001	0.742** 0.001	0.766** 0.001	0.614** 0.001	0.789** 0.001	1

** Significant at a level less than 0.01

To determine the reliability of the questionnaire, Cronbach’s alpha was calculated for the total scale (α=0.77) (Table 2). In order to evaluate the internal reliability of ISI, the Cronbach’s alpha was calculated by re-test method, which was 0.84 for the total index. In addition, 10% of the sample group (70 participants) were examined in terms of insomnia severity index 4-6 weeks later. The results of Spearman’s correlation test showed that the correlation coefficient was 0.88, which meant the required reliability was met.

**Figure 1:**
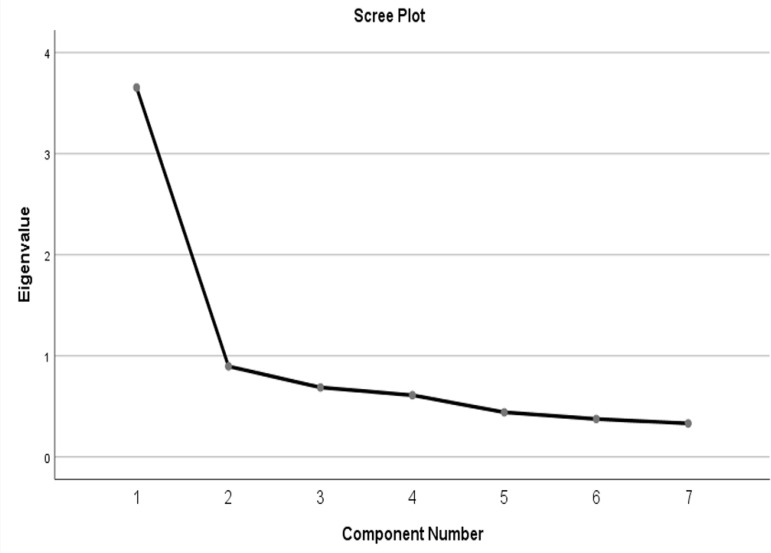
The scree plot of the number of factors of ISI.

Table 3 lists the variance, the cumulative percentage of variance, and the Eigen value of each item. According to the findings, the ISI contains seven items and one factor with an appropriate explanatory power with Eigen values >1. The scree plot shows the number of factors of ISI (Figure 1).

**Table 3:** Explained variance (%), cumulative variance (%) and Eigen value of factors of ISI and CFA of adolescent ISI.

Items	Eigen value	Explained variance (%)	Cumulative variance (%)	T-value (cr)	p-value
1	3.65	52.20	52.20	12.65	0.001
2	0.897	12.80	65.10	15.61	0.001
3	0.688	9.82	74.84	11.67	0.001
4	0.611	8.72	83.57	14.83	0.001
5	0.442	6.31	89.88	14.56	0.001
6	0.376	5.37	95.25	8.99	0.001
7	0.322	4.74	100	15.75	0.001

The results of CFA showed the ISI has suitable model for the adolescent population. The mean score of questions ranged from 0.96 to 1.63, and the value of T ranged from 8.99 to 15.75. Therefore, given the mean and the value of T, the questions are in the right range (Table 3).

Goodness of fit index (GFI) is well suited for values more than 0.9. In the developed model, the GFI value was 0.99, indicating goodness fit of the model. The closer confirmatory fit index (CFI) from 1, the more acceptable the model. In the model developed in this study, GFI was equal to 0.99 and acceptable. Bentler-Bonett normed fit index (NFI) equal to 0.9 represents a good fit of the model. Here, NFI value was 0.98, indicating the suitability of the model. Tucker-Lewis index (TLI) is a non-normed fit index and it ranges from 0 to 1. In this model, the TLI was 0.98, indicating the acceptability of the model (Table 4).

**Table 4:** Fitting indexes of the model of ISI in adolescents.

Fitting index of the model	χ^2^/DF	DF	CFI	NFI	GFI	TLI	RMSEA	r^2^
Rate	2.67	8	0.99	0.98	0.99	0.99	0.049	0.99
Criterion	<5	-	>0.8	>0.08	>0.08	>0.08	>0.08	Close to 1
Interpretation	Good fitting	Good fitting	Good fitting	Good fitting	Good fitting	Good fitting	Good fitting	Good fitting

**Figure 2:**
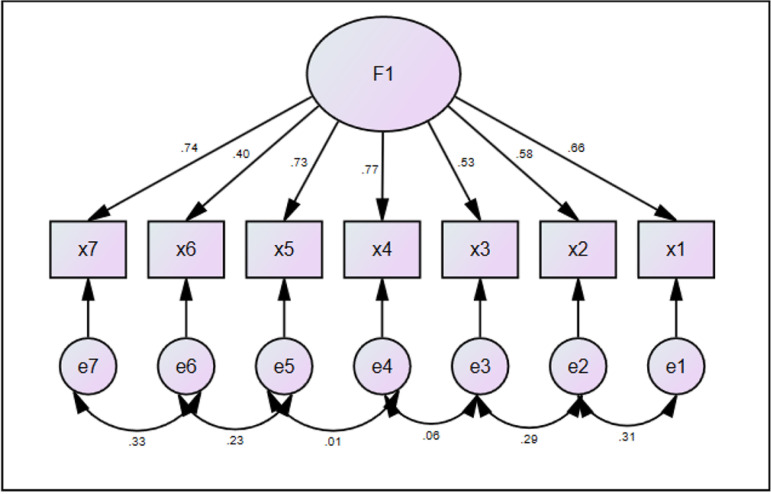
Single-factor model of insomnia severity index and its components in adolescents.

Root Mean Square Error of Approximation (RMSEA) is acceptable when it is less than 0.05 and indicates weakness of the model when it is higher than 0.11. The RMSEA value in this model was 0.049, which was in the range of 0.01 to 0.08 at a confidence interval of 90% and indicated the goodness of fit of the model (Table 4 and Figure 2).

Studying the concurrent validity of the ISI with other indexes showed that ISI had a direct and significant correlation with the total score of SQI (r=0.584, *p*-value=0.001), SHI (r=0.506, *p*-value=0.001), ESS (r=0.403, *p*-value=0.001) and GSAI (r=0.299, *p*-value=0.001), and all of which were significant at the level of 0.01 (Table 5).

**Table 5:** The correlation between ISI and indexes of daytime sleepiness, PSQI, SHI and GSAI in research units.

Index	Insomnia severity index	
	The correlation coefficient	The significance level
Pittsburgh sleep quality	**0.584	0.001
Sleep hygiene	**0.506	0.001
Epworth sleepiness	**0.403	0.001
Global sleep assessment	**0.299	0.001

** Significant at a level less than 0.01.

Moreover, Cronbach’s alpha was calculated to determine the reliability of the gender-based ISI and to determine the internal reliability of the ISI in female adolescents (α=0.78). The reliability of ISI was evaluated for 35 girls four to six weeks later. The Spearman’s correlation was equal to 0.85, which indicates a good reliability. Cronbach’s alpha was calculated to determine the reliability of the ISI in male adolescents (α=0.86). The reliability of ISI was evaluated in boys four to six weeks later and Spearman’s correlation was 0.89, which indicates a good reliability.

## DISCUSSION

The psychometric properties of the ISI were validated. The results showed that ISI had an acceptable reliability and validity for Iranian adolescents. The purpose of this study was to determine the reliability of the tool using the internal consistency method (Cronbach’s alpha). The results indicated that this index had a high internal consistency. Cronbach’s alpha coefficient was 0.77 for the total scale. In addition, Cronbach’s alpha, after about 4-6 weeks, was equal to 0.84, indicating a good reliability of this index. Bastien et al.^29^ calculated the construct validity of this test based on accuracy, severity, and satisfaction with a variance of 0.72 and its reliability was obtained based on Cronbach’s alpha equal to of 0.74 and 0.78, respectively. Castronovo et al.^30^ obtained the Cronbach’s alpha equal to 0.75, which was similar to the present study. Fernandez et al.^21^ also reported a good internal consistency (Cronbach’s alpha=0.82). Sadeghniiat et al.^18^ showed that the Persian version of the ISI had an acceptable internal consistency (Cronbach’s alpha=0.78). As noted in the findings, Eigen values were less than 1 in exploratory factor analysis, which explain the variance of variables. These findings indicate that the ISI with seven items can explain the severity of insomnia.

Another objective of this study was to determine the construct validity and factor structure using CFA and the results showed that a one-factor model was more suitable for the target group. Gerber et al.^17^ validated the German version of the ISI in adolescents, adults, and workers. The results of CFA showed that a one-way method was suitable for the model. In addition, the unchanging measurement among the genders was supported in all three samples, which is similar to the current study, and the both confirmed the fitness of the model^17^. The results of CFA for the Spanish version of the tool also confirmed the results of the present study^21^.

Another objective of this study was to determine the concurrent validity of the ISI with other indexes, and the results indicated a direct and significant correlation between this index and other indexes. Sadeghniiat et al.^18^ showed that the total score of ISI was significantly correlated with the total score of Pittsburgh sleep quality (*p*<0.001, r=0.74) and Beck Depression (*p*<0.001, r=0.42), while the total score for sleepiness scale was obtained as *p*<0.72 and r=0.12.

## CONCLUSION

The ISI had the sufficient reliability and validity for Iranian adolescent population and it can be used by future studies on Iranian community. It is suggested that this scale should be used in different ages, cultures, societies and genders in order to obtain a more accurate diagnostic value.

It is also suggested that the ISI as a tool for the diagnosis of sleep disorders should be used for adolescents with psychiatric disorders.
